# Mitochondria in health and disease: cellular powerhouses, signaling centers, and drivers of dysfunction

**DOI:** 10.3389/fcell.2026.1929130

**Published:** 2026-08-24

**Authors:** Olufemi Akintayo Akinkunmi, Feyikemi Funmilayo Araba, Jessica Adaeze Chukwudebelu, Victor Ademola Adenle, Ajibola Ayomide Odeyemi, Tunmise Maryanne Akhigbe, Roland Eghoghosoa Akhigbe

**Affiliations:** 1 Department of Physiology, Ladoke Akintola University of Technology, Ogbomoso, Oyo, Nigeria; 2 Reproductive Biology and Toxicology Research Laboratory, Oasis of Grace Hospital, Osogbo, Osun, Nigeria; 3 Department of Agronomy, Osun State University, Ejigbo, Osun, Nigeria

**Keywords:** mitochondria, mitochondrial dysfunction, mitochondrial transplantation, mitophagy, oxidative phosphorylation, ROS

## Abstract

Although the mitochondria are known as the cellular powerhouse, their function is beyond energy generation. These organelles regulate cellular metabolism, yet maintains a tightly regulated reactive oxygen species (ROS) generation and optimal redox state. In addition, mitochondria serve as mediators of physiological and pathological processes, such as maintenance of calcium balance, and control of apoptosis and mitophagy. All these make the mitochondria a major factor in both cellular and organismal regulation. However, mitochondria dysfunction may occur through many processes, including genetic mutations, increased production of ROS, metabolic failure from impaired electron transport chain activity, and dysregulated dynamics or mitophagy. Several self-perpetuating damages accumulate from these processes and influence clinical pathologies, such as aging, metabolic syndrome, cancer, neurodegeneration, and reproductive disorders. Recent studies demonstrate promising therapeutic targets for mitochondrial dysfunction. Examples include targeted antioxidants, such as MitoQ and SkQ1, to selectively neutralize mitochondrial ROS, pharmacological modulators to enhance mitochondrial biogenesis and to restore NAD^+^ homeostasis via PGC-1α activation, gene-editing technologies, such as mitoTALENs and mtZFNs to selectively eliminate pathogenic mitochondrial DNA mutations, and mitochondrial transplantation as a new technique to replace damaged organelles. Together, these novel approaches highlight the need for research in mitochondrial function to change the therapeutic landscape in the management of mitochondrial dysfunction-associated diseases.

## 1 Introduction

Mitochondria are evolutionarily old organelles, which originated 1.5 billion years ago from a free-living aerobic prokaryote engulfed by a primitive eukaryotic host cell, eventually giving rise to the modern mitochondrion (Hampl and Roger, 2024). The endosymbiotic theory, which first emerged in the 1960s from the work of Lynn Margulis (Gray, 2017), is now widely accepted and accounts for the double membrane, circular genome and prokaryotic-like ribosomes of mitochondria. Mitochondria have become integral components in cellular physiology, regulating energy metabolism, signaling, stress responses and cell fates, unlike in the past when they were viewed as simple sources of energy. (Chakrabarty and Chandel, 2021).

Mitochondria are known for their involvement in energy production through oxidative phosphorylation (OXPHOS) which takes place in the inner mitochondrial membrane, where electron transport chain (ETC) complexes I–IV generate a proton gradient which is then catalyzed by ATP synthase to produce ATP (Spinelli and Haigis, 2018). Mitochondria are also very flexible metabolically, satisfying the specific energy needs of the tissue either by oxidizing glucose-derived pyruvate, fatty acids via β-oxidation, or amino acids (Inigo et al., 2021; Panov et al., 2024). This metabolic flexibility allows for cellular homeostasis in any energy source (Vyas et al., 2016). Moreover, mitochondria regulate calcium handling through mitochondrial calcium uniporter (MCU) (Rossi et al., 2019), to produce ROS which are signaling molecules at physiological levels (Murphy, 2009), and to induce intrinsic apoptosis in pathological conditions through the release of cytochrome c (Czabotar and Garcia-Saez, 2023). These functions make maintenance of mitochondrial integrity, through coordinated dynamics (fusion/fission), mitophagy, and cristae remodeling, important for cellular health (Cogliati et al., 2016; Chan, 2020).

Mitochondrial dysfunction resulting from bioenergetic failure, gene mutations, or oxidative stress is associated with a broad range of human pathologies including metabolic syndrome, neurodegenerative conditions, cancer, and reproductive pathologies among others (Zong et al., 2024; Akhigbe and Hamed, 2021; Lasisi-Sholola et al., 2024). Thus, it is fundamental to understand how these mechanisms maintain mitochondrial function in order to identify strategies to restore cellular and organismal balance for therapeutic purposes.

## 2 Mitochondrial function in cellular homeostasis

**FIGURE 1:**
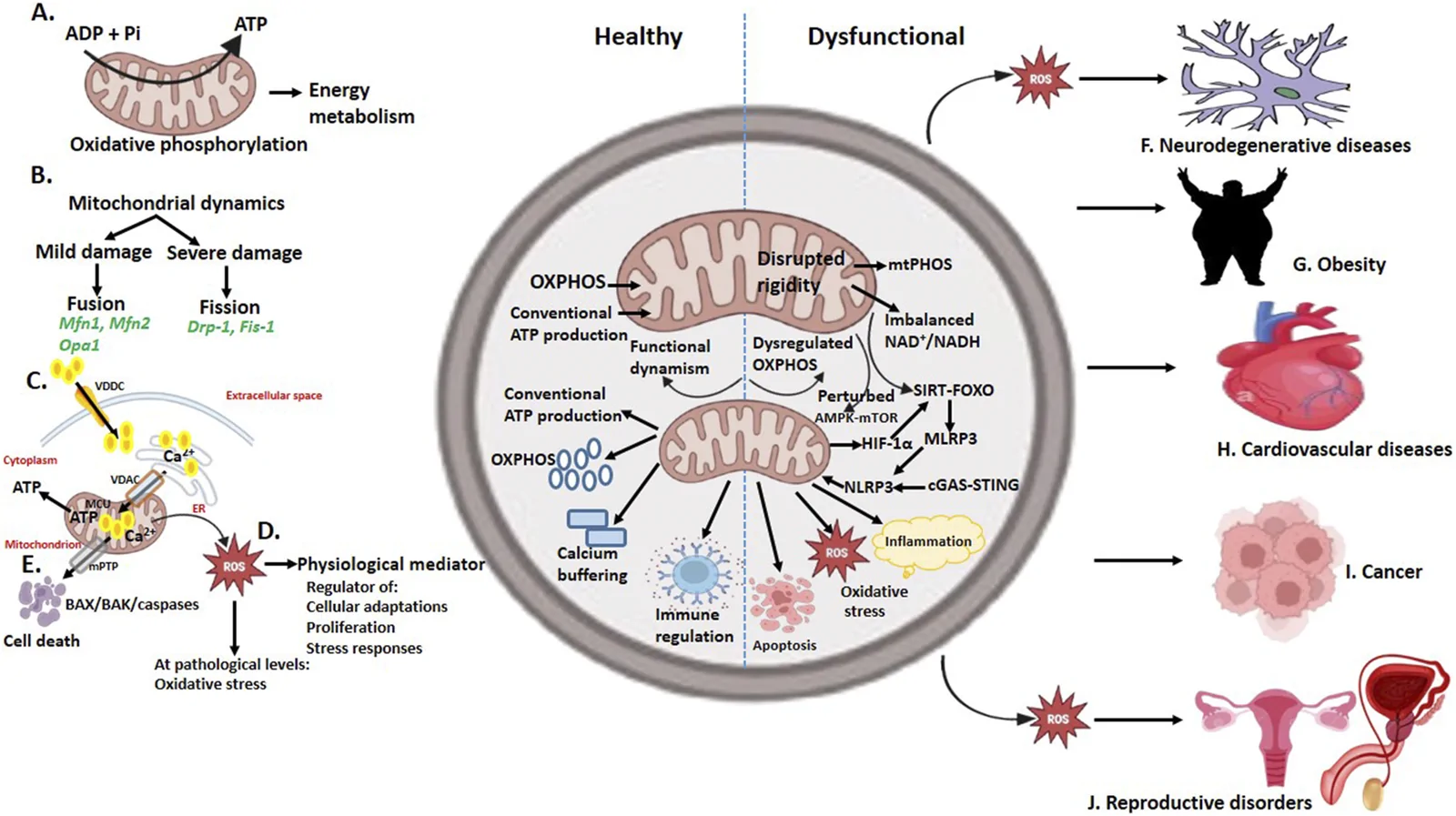
Mitochondrial function in cellular homeostasis and pathological. Mitochondria maintain cellular homeostasis via **(A)** the regulation of energy metabolism through oxidative phosphorylation (OXPHOS), substrate flexibility and metabolic integration **(B)** maintenance of structural and functional integrity through fission and fusion that is facilitated by mitofusins (MFN1/2) and optic atrophy protein 1 (OPA1), and dynamin-related protein 1 (DRP1) and fission 1 protein (FIS-1) respectively, **(C)** promotion of calcium influx, **(D)** and generation of ROS generation, which mediate cellular adaptation, proliferation and stress response but at pathological levels could induce oxidative stress. **(E)** By modulating cytochrome c/BAX/BAK/caspases, it also balances cell survival and cell death. However, mitochondria dysfunction leads to **(F)** neurodegenerative disease, **(G)** obesity, **(H)** cardiovascular disease, **(I)** cancer, and **(J)** reproductive disorders.

Mitochondria maintain cellular homeostasis via oxidative phosphorylation, substrate flexibility, calcium buffering, ROS signalling, and apoptosis regulation through coordinated fusion/fission dynamics and mitophagy. However, mitochondrial dysfunction from genetic defects (mtDNA and nDNA mutations), bioenergetic failure, oxidative stress, impaired dynamics, and NAD^+^/NADH redox imbalance interact in self-perpetuating cycles, involving the induction of mtDNA damage by ROS, which impairs, ETC., function, generating more ROS and bioenergetic collapse. Disrupted mitophagy and calcium handling further exacerbate cellular injury, establishing mitochondria as central drivers of pathogenesis across multiple diseases, such as neurodegenerative, metabolic, and cardiovascular diseases, as well as cancer, reproductive disorders, and age-related disorders (Figure 1).

### 2.1 Energy metabolism

The mitochondria play a central role in regulating energy metabolism, through oxidative phosphorylation (OXPHOS), substrate flexibility and metabolic integration (Leverve, 2007). Oxidative phosphorylation takes place in the inner mitochondrial membrane where electrons derived from NADH and FADH_2_ pass through the electron transport chain (ETC) complexes I–IV, generating a proton gradient that drives ATP synthesis via ATP synthase (Complex V). This process links the use of oxygen with ATP generation and it is needed in maintaining energy balance (Spinelli and Haigis, 2018). The mitochondria are flexible in utilization of substrates; hence, they can adapt to several nutrient availability. Pyruvate is converted to acetyl-CoA and then enters tricarboxylic acid (TCA) cycle. In high-energy demanding tissues like cardiac and skeletal muscle, β-oxidation of fatty acids generates substantial ATPs, alongside the production of NADH and FADH_2_ (Panov et al., 2024). Amino acids are also another source of metabolic substrates for the TCA cycle, serving as anaplerotic metabolites (Inigo et al., 2021). The metabolic plasticity helps mitochondria maintain energy homeostasis in response to physiological stress and fluctuations in nutrients (Vyas et al., 2016; Spinelli and Haigis, 2018). Mitochondria also regulate biosynthetic pathways and redox balance, that influence cellular proliferation and differentiation (Antico Arciuch et al., 2012). Alterations in mitochondrial bioenergetics are linked to metabolic disorders (Akhigbe and Hamed, 2021; Pietrangelo et al., 2025), reproductive dysfunction (Akhigbe et al., 2025), cancer, and neurodegenerative diseases (De Moura et al., 2010; Tassone et al., 2023; Lasisi-Sholola et al., 2024); this highlights the role of mitochondria in cellular homeostasis.

### 2.2 Mitochondrial dynamics

Mitochondria are dynamic networks that undergo fission and fusion continuously for maintaining their structural and functional integrity. Fusion, facilitated by mitofusins (MFN1/2) and optic atrophy protein 1 (OPA1), allows the interaction between the content of the mitochondria, such as mitochondrial DNA (mtDNA) and metabolic enzymes (Ranieri et al., 2013). Mitochondrial segregation and quality control (mitochondrial QC) is a process regulated primarily by a process called fission, which is regulated by dynamin-related protein 1 (DRP1) (Chan, 2020). Cristae remodeling is also a part of mitochondrial dynamics. Cristae are folds of inner membrane containing the respiratory chain complexes. The remodeling of cristae structure influences the efficiency of respiration and apoptotic signal transduction by controlling the sequestration of cytochrome c (Cogliati et al., 2016). Loss of cristae structure may affect OXPHOS and enhance the vulnerability to apoptosis.

The removal of damaged organelles and mitochondrial turnover are maintained by selective autophagy, known as mitophagy (Uoselis et al., 2023). Mitophagy prevents accumulation of dysfunctional mitochondria and excessive reactive oxygen species (ROS), thereby maintaining cellular homeostasis (Pickles et al., 2018). The bioenergetic adaption and apoptotic sensitivity are thus regulated by mitochondrial dynamics.

### 2.3 Calcium handling and signaling

Mitochondria play a central role in intracellular calcium (Ca^2+^) homeostasis. The mitochondrial calcium uniporter (MCU) complex is the major mechanism for calcium uptake, and is activated by the mitochondrial membrane potential. This uptake regulates the activity of enzymes involved in the TCA cycle, increasing ATP production when the cell requires more (Rossi et al., 2019).

Mitochondria and endoplasmic reticulum (ER) interactions are facilitated by mitochondria-associated membranes (MAMs), enabling the rapid movement of calcium ions (Ca^2+^) from the ER to the mitochondria; a process that coordinates energy production with cellular signaling events (Marchi et al., 2017). A proper ER–mitochondria coupling ensures efficient Ca^2+^ buffering and prevents cytosolic calcium overload (Giorgi et al., 2015). However, excess Ca^2+^ accumulation in the mitochondrial can trigger opening of the mitochondrial permeability transition pore (mPTP), leading to membrane depolarization and cell death (Morciano et al., 2021a). Therefore, the regulation of mitochondrial calcium serves as cellular bridge that directly link energy metabolism with cell survival.

### 2.4 ROS generation and antioxidant defense

ROS are generated naturally during mitochondrial respiration, mainly at complexes I and III of the, ETC. Moderate ROS plays an important role in the regulation of cellular adaptations, proliferation and stress responses, whereas excess ROS is potentially harmful to lipids, proteins and DNA (Murphy, 2009; Sena and Chandel, 2012; Akhigbe, 2025).

The dual signaling/damage function of ROS emphasizes the importance of mitochondrial antioxidant systems. Superoxide dismutase (SOD) converts superoxide into hydrogen peroxide, which is further detoxified by catalase and glutathione peroxidase. At physiological concentrations, ROS can act as crucial mediators of redox signaling, while excess ROS can cause oxidative stress and result in various diseases such as aging and neurodegeneration (Shadel and Horvath, 2015). Thus, mitochondrial ROS can serve as intercellular messengers as well as potential mediators of cellular damage if antioxidants are compromised.

### 2.5 Apoptosis and cell death

Mitochondria are central regulators of intrinsic (mitochondrial) apoptotic pathways. When the cell is stressed, proapoptotic Bcl-2 family proteins (such as BAX and BAK) facilitate the permeabilization of the outer membrane, allowing the release of cytochrome c into the cytosol of the stressed cell (Czabotar and Garcia-Saez, 2023). Apoptotic protease activating factor-1 (Apaf-1) interacts with the cytochrome c to form the apoptosome and activate caspase-9, leading to the activation of downstream caspases (Wang et al., 2020).

Mitochondrial architecture is linked to the regulation of apoptosis by the remodeling of cristae, which is involved in the mobilization of cytochrome c from intracristal spaces (Ježek et al., 2023). Furthermore, mitochondrial permeability transition also links to necrotic and apoptotic cell death in cases of calcium overload and oxidative stress (Morciano et al., 2021a). By controlling the release of cytochrome c and the activation of caspases, mitochondria are important decision makers that maintain tissue homeostasis through balancing cell survival and cell death.

## 3 Mechanisms of mitochondrial dysfunction

Mitochondrial dysfunction can be defined as the impairment of some key mitochondrial functions, such as oxidative phosphorylation, redox regulation, metabolic integration, calcium homeostasis, and organelle quality control. The definition firstly emerged in the context of bioenergetic concepts, but today, it is a wider description of the overall purpose of mitochondria as biosynaptic and signaling platforms in the cellular environment (Zong et al., 2024). Primary mitochondrial diseases can cause dysfunction that is induced by inherited mutation in mitochondrial DNA (mtDNA) or in nuclear-encoded mitochondrial genes, whereas non-mitochondrial diseases may cause dysfunction as a secondary complication of such common pathological conditions as neurodegeneration, cardiovascular disease, and metabolic disorders (Zong et al., 2024; Khan et al., 2022). Usually, mitochondrial impairment is multifactorial; most abnormal bioenergetics, high levels of ROS production, abnormal dynamics, mitophagy, and calcium balance often face each other and interact in self-positive cycles (Salis Torres et al., 2024; Yusuf et al., 2025). All these interdependent processes impair cellular homeostasis and promote disease pathogenesis.

### 3.1 Genetic defects

Mitochondrial diseases are an eclectic category of genetic disorders that are caused by defects that affect mitochondrial functioning. The defects can be caused by mutations in mitochondrial DNA (mtDNA) or nuclear DNA (nDNA) that encode proteins involved in oxidative phosphorylation (OXPHOS) and more extensive mitochondrial homeostasis (Stenton and Prokisch, 2020; Wen et al., 2025). Even though mitochondrial proteins are predominantly nuclear-encoded, despite only 13 structural subunits of the respiratory chain being coded by mitochondrial DNA, the dual genomic regulation of mitochondrial activity is clear (Alston et al., 2017; Stenton and Prokisch, 2020). In contrast to diploid nuclear DNA, the mitochondrial DNA is inherited maternally and is present in more than one copy per cell, which results in heteroplasmy, the presence of mutant and wild-type cytoplasmic mitochondrial DNA (mtDNA) in the same cell (Parakatselaki and Ladoukakis, 2021). mtDNA mutations that can be in the form of point mutation and extensive deletions and are associated with classic syndromes such as mitochondrial encephalomyopathy, lactic acidosis, and Stroke-like episodes (MELAS), Kearns-Sayre syndrome, and chronic progressive external ophthalmoplegia (Alston et al., 2017).

However, nuclear-encoded genes of the mitochondrion are inherited according to the patterns of Mendelian inheritance, such as autosomal recessive, autosomal dominant, X-linked, or *de novo* inheritance, and can lead to the impairment of mitochondrial respiratory chain assembly, maintenance of the mitochondrial DNA, and protein translation (Stenton and Prokisch, 2020; Wen et al., 2025). All these genetic malformations impair the efficiency of OXPHOS and cause subsequent bioenergetic imbalances, leading to mitochondrial dysfunction.

### 3.2 Bioenergetic failure

ATP production primarily occurs in the mitochondria, where an oxidative phosphorylation (OXPHOS) process occurs, and it includes electron transfer via the respiratory chain to protons translocation across the inner mitochondrial membrane. Complexes I-IV sequentially transfer the electron donors (NADH and FADH2) to produce a proton motive force to generate ATP by Complex V (ATP synthase) (Wallace et al., 2010). Impaired, ETC., which is common in pathological conditions, decreases the availability of ATP in cells. As an outcome of mutations in OXPHOS subunits, impaired assembly of respiratory complexes, or disorganization of respiratory super complexes, these affect the stability of electron flow and reduce the formation of proton gradients (Nesci and Lenaz, 2022; Zong et al., 2024). These disruptions lead to ineffective oxidative phosphorylation and reduced ATP synthesis and, therefore, affect energy-intensive activities such as ion transportation, biosynthesis, and cellular signaling.

Besides decreased ATP synthesis, ETC., malfunction facilitates electron leakage especially at Complex I and Complex III, making the mitochondria even more unstable (Wallace et al., 2010). The resultant bioenergetic disequilibrium compels cells to move to less efficient metabolic activities like glycolysis that is not able to completely replace the mitochondrial ATP production that is disrupted. Prolonged energetic failure eventually leads to the disruption of calcium, membrane potential, and the activation of cell death mechanisms (Zong et al., 2024; Miller et al., 2025). Altogether, bioenergetic failure is one of the key processes of mitochondrial dysfunction, which connects the structural abnormalities of the respiratory chain with the systemic metabolic disturbances that are known to be present in a wide range of diseases.

### 3.3 Oxidative stress and damage

ROS include free radical species like superoxide (O_2_^−^) and hydroxyl radical (OH), and non-radical oxidants like hydrogen peroxide (H_2_O_2_). They act as signaling molecules that control cell proliferation, differentiation and cellular responses to stress in physiological concentrations (Averill-Bates, 2024). Nevertheless, excessive production of ROS overloads the endogenous antioxidants and leads to oxidative stress (Venditti and Di Meo, 2020; Juan et al., 2021).

Mitochondria represent one of the largest sources of intracellular ROS because of leakage of electrons through, ETC., especially at Complex I and Complex III. In a basal condition, about 1%–2% of ingested oxygen can be turned to superoxide and quickly dissolved by mitochondrial superoxide dismutase to H_2_O_2_ (Murphy, 2009; Venditti and Di Meo, 2020). ETC impairment enhances electron leakage in pathological conditions, like hyperglycemia, hypoxia, or ischemia-reperfusion injury, with a significant contribution to the production of ROS (Zhang et al., 2023). Overproduction of mitochondrial ROS induces lipid, protein and nucleic acid damage. Mitochondrial DNA (mtDNA) is especially susceptible as it is closer to the ETC, has no protection histones, and the capacity to repair it is relatively low (Chinnery et al., 2012). Transcription of respiratory chain elements and additional damage to ATP production are caused by oxidative changes like 8-oxo-7,8-dihydroguanine (8-oxoG), strand breaks, and crosslinks of the mitochondrial DNA with proteins (Juan et al., 2021; Xu et al., 2025). DNA polymerase gamma (POLG) oxidative damage also leads to decreased fidelity of replication of the mitochondrial genome, and more mutational load and genomic instability (Xu et al., 2025).

Oxidative stress creates its own self-perpetuating feedback loop. mtDNA mutation induced by ROS alters the efficiency of ETC that increases the leakage of electrons and more generation of ROS. Bioenergetic failure is aggravated by concurrent oxidative inactivation of enzymes of the respiratory chain and Krebs cycle components (Murphy, 2009; Xu et al., 2025). When the extent of oxidative damage is great, mitochondrial membrane potential falls, leading to opening of permeability transition pore (PTP), release of cytochrome c, and induction of apoptotic pathways (Murphy, 2009). Irrespective of the existence of strong antioxidant systems in mitochondria and certain adaptive mechanisms to eliminate all damaged organelles by means of mitophagy, chronic oxidative stress affects mitochondrial homeostasis and promotes the development of dysfunctional cells (Venditti and Di Meo, 2020; Xu et al., 2025). Therefore, oxidative stress is one of the primary mechanisms that interconnect mitochondrial damage, genomic instability, dysfunctional bioenergetics, and cell death under varying pathologic conditions.

### 3.4 Altered dynamics and quality control

Mitochondrial homeostasis requires fusion, fission and mitophagy to ensure the integrity and bioenergetic competence of the mitochondria. The outer and inner membrane of the mitochondrion are fused by mitofusin-1/2 (MFN1/2) and optic atrophy protein-1 (OPA1), to allow fusion of mitochondrial DNA, proteins, and metabolites to dilute local injuries and protect the functionality (Chan, 2020; Chen et al., 2023). OPA1 regulates cristae morphology and respiration efficiency, underlining its key role in maintaining oxidative phosphorylation (Chen et al., 2023). Mitochondrial fission, in contrast, is regulated by dynamin-related protein-1 (Drp1) which is translocated to the outer mitochondrial membrane by adaptor proteins such as mitochondrial fission factor (Mff), fission 1 protein (Fis1), mitochondrial dynamics protein of 49 kDa (MiD49), and mitochondrial dynamics protein of 51 kDa (MiD51) (Zerihun et al., 2023). Post-translational modifications such as phosphorylation, Small Ubiquitin-like Modifier (SUMOylation), ubiquitination and S-nitrosylation modification are important regulators that help in the activation of Drp1 to enable modulation of mitochondrial division in a signal-dependent manner (Scheffer et al., 2022; Chen et al., 2023).

Though mitochondrial distribution and quality control is achieved through controlled fission, excessive Drp1 activation induces fragmentation, membrane depolarization, and bioenergetic collapse (Chan, 2020; Scheffer et al., 2022). MFN or OPA1 dysfunction fusion also leads to fragmented cristae structure, low production of ATP, and the elevated vulnerability to undergo apoptosis (Chen et al., 2023). Mitophagy is a process which is a complementary quality control mechanism as it selectively removes dysfunctional mitochondria (Pickles et al., 2018). PINK1 (PTEN-induced kinase 1) or Parkin pathway is a ubiquitin-independent channel of dysfunctional mitochondria recognition, which involves the recruitment of autophagy adaptors and the generation of autophagosomes (Wang et al., 2021; Uoselis et al., 2023). This surveillance system is further extended by receptor-mediated pathways (Uoselis et al., 2023). Mitochondrial dysfunction results in oxidative stress, and increases the susceptibility to incident neurodegenerative and cardiovascular pathologies (Wang et al., 2021; Scheffer et al., 2022). Basically, mitochondrial fission, unsuccessful fusion and mitophagy defects all lead to the impairment of mitochondrial network stability to cause progressive and chronic cell dysfunction.

### 3.5 Impaired signaling and metabolite imbalance

Mitochondrial dysfunction is known to cause cellular homeostasis disruption not just by the bioenergetic failure but also by the distortion of cellular metabolic signaling. The focus of this is the disequilibrium of the NAD^+^/NADH redox couple. NAD^+^ is vital to oxidative metabolism, and its degradation, along with the accumulation of NADH, is an indicator of dysfunction in the activity of the electron transport chain and the promotion of reductive stress (Amjad et al., 2021; Mercola, 2025). An increased NADH: NAD^+^ ratio increases reverse electron transfer and superoxide formation, which connects metabolic derangement to oxidative damage and inflammation (Mercola, 2025). Age-related NAD + depletion also leads to mitochondrial dysfunction-related senescence and pro-inflammatory signaling (Somasundaram et al., 2024).

In addition to metabolism, NAD^+^ controls mitochondrial quality control by its NAD^+^- sensitive enzymes like sirtuins (Anderson et al., 2017). Specifically, Mitochondrial sirtuin 3 (SIRT3) regulates antioxidant defenses, mitochondrial dynamics, mitophagy and the mitochondrial unfolded protein response (UPRmt) (Zong et al., 2024; Yusri et al., 2025). A decrease in the bioavailability of NAD + suppresses these protective mechanisms, thus increasing oxidative stress and dysfunction of organelles.

Mitochondria have a lot of organelle interaction as well. Communication with the endoplasmic reticulum regulates the flow of calcium and lipid exchange, and the lysosomal activity regulates the flow of iron homeostasis and autophagy (Diogo et al., 2018; Audano et al., 2020; Heiby and Ori, 2022). The disturbance of these signaling networks results in impaired ATP production and promotion of apoptotic and inflammatory processes. The metabolic imbalance and the disrupted inter-organellar cross-talk change the mitochondria into the drivers of cellular destruction or degeneration.

## 4 Mitochondrial dysfunction in disease

Mitochondria play an essential role in modulating cellular bioenergetics, intracellular interactions, and redox homeostasis (Schirrmacher, 2020). Although mitochondria are known for their conventional ATP production, their functional dynamism extends to several physiological functions, including OXPHOS, calcium buffering, modulation of apoptotic responses, regulation of cellular metabolites and stem cell renewal, immune regulation, and metabolic adaptation (Yan et al., 2021). However, impaired mitochondrial homeostasis and bioenergetics have been implicated in several pathogenic conditions, disrupting OXPHOS, mitochondrial dynamics regulation, mtDNA rigidity, mitophagy, and NAD^+^/NADH redox balance through perturbed AMPK-mTOR, NF-κB, SIRT-FOXO, HIF-1α, cGAS-STING, and NLRP3 inflammasome pathways (Poljšak and Milisav, 2024). Thus, mitochondrial dysfunction is associated with metabolic derangement, inflammation, oxidative stress, and cell death, all of which are drivers of human pathological diseases (Jurcău et al., 2022; Yousef et al., 2023).

### 4.1 Neurodegenerative diseases

Neuronal cells, being highly metabolic cell, relies majorly on mitochondrial output and OXPHOS to enhance synaptic transmission (Li and Sheng, 2021). Altered complex I expressions promote neuronal susceptibility to excitotoxicity by upregulating superoxide generation and disrupting ATP-dependent ion gradient (Verma et al., 2022). Also, impaired mitophagy activity initiated by altered PINK1/Parkin-mediated ubiquitination of mitochondrial outer membrane proteins suppresses the removal of depolarized mitochondria, exacerbating oxidative stress and mtDNA damage in dopaminergic neurons (Barazzuol et al., 2020). Furthermore, mitochondrial calcium dysregulation via mitochondrial calcium uniporter (MCU) aggravates excitatory glutamatergic signaling, thus promoting mPTP opening and the release of cytochrome c, while transmutations in TDP-43, SOD1, and C9orf72 distort mitochondrial histoarchitecture (cristae structure), disrupting mitochondrial supply, and initiating mPTP opening (Barazzuol et al., 2020).

Nonetheless, a simultaneous decrease in NAD^+^ level suppresses ROS detoxification by downregulating SIRT3-mediated deacetylation of mitochondrial antioxidant enzymes, including superoxide dismutase II, all of which collectively promote apoptosis in the motor neurons (Khan et al., 2021). However, impaired mitochondria also activate cGAS-STING signaling and downstream NF-κB-dependent neuroinflammatory responses through the release of mtDNA into the cytosol, while concurrent activation of NLRP3 inflammasome further promotes IL-1β production, thereby exacerbating neurodegenerative signaling cascades (Jurcău et al., 2022). These molecular alterations highlight the role of mitochondria and their regulation in neurodegenerative diseases, such as Parkinson’s and Alzheimer’s disease (Jurcău et al., 2022).

### 4.2 Metabolic disorders

Chronic positive energy gain observed in obesity and diabetes promotes electron flux by engulfing, ETC., capacity, thereby upregulating mitochondrial ROS production, enhancing inflammation in the adipose tissue, and disrupting redox homeostasis (Corkey, 2023). The elevated ROS mediate JNK/IKKβ activation stimulates serine phosphorylation of IRS protein, downregulating P13K/AKT signaling and altering insulin sensitivity (Qin et al., 2025). However, reduced stimulation of AMPK signaling downregulates PGC-1α-dependent mitochondrial biogenesis, inhibits endogenous oxidative capacity and enhances the accumulation of lipid intermediates, such as diacylglycerols and ceramides, which activate PKC isoforms, further promoting insulin resistance by downregulating insulin receptor signaling (Chen et al., 2025).

Furthermore, impaired interaction between glycolysis and OXPHOS in the pancreatic β-cells disrupts ATP/ADP ratio, altering the closure of K/ATP channels and Ca^2+^-mediated insulin exocytosis (Mu-U-Min et al., 2023). Also, increased mitochondrial ROS activates FOXO1 and β-cells phenotypic regression, while NAD^+^ depletion perturbs SIRT1/SIRT3 expression, suppressing mitochondrial antioxidant activity and metabolic flexibility, thus highlighting the role of mitochondria in metabolic regulations (Fan et al., 2020). Nonetheless, utilization of AMPK activators, sirtuin modulators, or NAD^+^ precursors has been shown to restore mitochondrial redox homeostasis and metabolic flexibility, accentuating the mitochondrial restoration approach as a potential therapeutic paradigm in metabolic diseases (Palabiyik and Palabiyik, 2025).

### 4.3 Cardiovascular diseases

Cardiac tissue known for its high metabolic demand, shows high mitochondrial density, rendering it particularly vulnerable to impaired bioenergetics (Morciano et al., 2021b). Reverse electron transport at complex I observed in ischemia-reperfusion produces eruption of ROS, triggering the opening of mPTP through the cyclophilin D-mediated pathway, thereby disrupting mitochondrial histology, inhibiting ATP production and activating necrotic cell death (Pietrangelo et al., 2025). However, increased DRP1-mediated mitochondrial fission promotes myocardial cell apoptosis through Oligomeric activation of BAX/BAK, and membrane permeabilization, while impaired mitophagy simultaneously leads to the build-up of dysfunctional mitochondria that aggravates oxidative stress (Tong et al., 2020). Furthermore, efflux of mtDNA into the circulation initiates TLR9 and cGAS/STING signaling pathways, triggering inflammatory responses mediated by NF-κB activation, which promotes atherosclerosis, while activation of NLRP3 inflammasome further exacerbates sterile inflammation in myocardial injury (Pietrangelo et al., 2025).

### 4.4 Cancer

Alterations in enzymes, including fumarate hydratase (FH) and succinate dehydrogenase (SDH), that are involved in the TCA cycle lead to the accumulation of fumarate, succinate, and 2-hydroxyglutarate (2-HG), which downregulate α-ketoglutarate-dependent dioxygenases (Sarkar et al., 2025). The inhibition of α-ketoglutarate-dependent dioxygenases modulates HIF-1α, disrupting histone and DNA methylation, thus shifting the epigenetic landscape towards a tumor-friendly microenvironment (Xiong et al., 2023; Sarkar et al., 2025). However, mitochondrial folate cycle promotes the biosynthesis of nucleotides and NADPH, enhancing the GSH-dependent antioxidant defense mechanism (Marí et al., 2009). Impaired mtROS signaling cascades activate NF-κB and PI3K/AKT/mTOR pathways, thereby promoting tumor proliferation, survival and metabolic flexibility (Lin et al., 2024) by increasing the ability to adapt to harsh microenvironments through epithelial-to-mesenchymal transition (EMT) and increased autophagy (Du et al., 2025). The persistent activation of these pathways by mtROS is a primary mechanism that drives therapy resistance, as these signaling networks allow cells to avoid apoptosis, even under pharmacological stress (Jiang et al., 2025). Furthermore, overexpression of anti-apoptotic Bcl-2 family protein leads to apoptotic resistance and inhibit BAX/BAK via the formation of a high-affinity binding sink that sequesters pro-apoptotic BH3-only proteins (Rooswinkel et al., 2014; Qian et al., 2022). This inhibits the generation of BAX/BAK oligomeric pores on the mitochondrial outer membrane (MOMP), which normally release cytochrome c and trigger caspase activation (Danial, 2007). Inhibition of mitochondrial-mediated apoptosis promotes cell survival in cellular stress, resulting in accumulation of oncogenic mutations and tumor development and therapeutic resistance (Qian et al., 2022). The elevated levels of Bcl-2 proteins permit tumor cells to survive in low-nutrient environments, resist chemotherapeutic drugs, and avoid DNA damage-driven cell death (Montgomery-Song, 2022), while the high expression of anti-apoptotic members, often caused by chromosomal translocations or amplification, contributes directly to the development of lymphomas and other cancers (Vega et al., 2002; Carneiro and El-Deiry, 2020). Disrupted mitochondrial dynamics, particularly DRP1-mediated fission, promote metastatic capacity via cytoskeletal remodeling and metabolic flexibility (Lin et al., 2024). Upregulation of DRP1, usually induced by oncogenic signaling (such as KRas, ERK) and post-translational modifications (phosphorylation at Ser616), fragments mitochondria, which promotes cancer cell invasion, migration, and survival (Mishra et al., 2025).

### 4.5 Reproductive disorders

Reproductive organs possess a high energy demand for gametogenesis, steroidogenesis and early embryonic development, possibly due to complex molecular, hormonal and enzymatic activities (Aitken et al., 2022). Impaired mitochondrial membrane potential and elevated ROS generation in the testicular cells stimulate intrinsic apoptotic signaling through the release of cytochrome c, thereby disrupting spermatogenic and steroidogenic activities, leading to impaired sperm DNA and overall sperm quality, ensuing male infertility (Fadel et al., 2020). In polycystic ovarian syndrome (PCOS), mitochondrial dysfunction activates JNK and NF-κB signaling pathways due to increased ROS generation, triggering chronic low-grade inflammation and reduced follicular growth, while impaired AMPK/mTOR signaling disrupts metabolic sensitivity within the ovarian granulosa cells (Ouyang et al., 2025; Voros et al., 2026).

However, metabolic plasticity observed in ovarian cancer enhances dynamic changes between glycolysis and OXPHOS (Kobayashi, 2022). The metabolic plasticity allows ovarian tumor cells to dynamically switch between glycolysis and OXPHOS and permit adaptation to environmental stresses, such as hypoxia, nutrient deprivation, and therapeutic pressure (Keoh et al., 2025). This ability to rewire energy metabolism is a key driver of metastasis, chemoresistance, and recurrence (Keoh et al., 2025; Liu et al., 2025). Initially, ovarian cancer cells depend on glycolysis, but often switch to OXPHOS-dominant metabolism to survive chemotherapy; thus, allowing resistant cells to produce ATP more efficiently and manage increased oxidative stress through robust mitochondrial function.

Although endometriotic lesions demonstrate metabolic reprogramming and mitophagy with increased OXPHOS and ROS generation, and promote ectopic cell survival in hypoxic condition, increased mtROS further enhance the maintenance of HIF-1α and angiogenic signaling cascades (Kim et al., 2025). Endometriotic lesions undergo metabolic reprogramming characterized by a paradoxical upregulation of OXPHOS despite their hypoxic microenvironment (Ng et al., 2020). This shift is mediated by estrogen receptor beta (ER-β) signaling and PGC-1α-mediated mitochondrial biogenesis that elevates mitochondrial density and increases oxygen utilization efficiency (Kobayashi et al., 2023). Nonetheless, enhanced OXPHOS activity triggers elevated mtROS generation from, ETC (Huang et al., 2025). Concomitantly, these lesions show dysregulated mitophagy via the PINK1/Parkin pathway and BNIP3 activation, which selectively inhibits dysfunctional mitochondria while retaining those with functional, ETC.,; thus, sustaining ATP production and preventing mitochondrial dysfunction (Zhao et al., 2024). This causes elevated mtROS state, upregulates mitochondrial antioxidant defenses, and promotes cell resistances; these mtROS also serve as signaling molecules rather than just being cytotoxic byproducts (Huang et al., 2025).

The elevated mtROS directly stabilize HIF-1α via oxidative inactivation of prolyl hydroxylases and the VHL ubiquitin ligase, inhibiting HIF-1α degradation even under conditions where oxygen tension would normally permit it (Zhang J. et al., 2025a). Stabilized HIF-1α moves to the nucleus and promotes pro-angiogenic factors transcription, including VEGF and angiopoietins, initiating an angiogenic cascade to the lesion (Chung and Han, 2022). This neovascularization promotes the supply of oxygen and iron, which further fuels OXPHOS activity and generates additional ROS via Fenton reaction, leading to a self-perpetuating cycle where metabolic adaptation, mitochondrial quality control, ROS signaling, and angiogenesis reinforce one another to promote ectopic cell survival, proliferation, and lesion maintenance despite the hostile hypoxic environment (Perrone et al., 2025).

### 4.6 Aging and age-related diseases

Aging can be categorized as a progressive mitochondrial and molecular alteration, including overall mtDNA changes, impaired mitophagy, reduced mitochondrial bioenergetics and cellular NAD^+^ (Amorim et al., 2022). These progressive alterations occur as a result of impaired AMPK–PGC-1α signaling pathways, disrupting mitochondrial bioenergetic activity and metabolic regulations, leading to increased mtROS production and reduced OXPHOS capacity (Vernier and Giguère, 2021). These subsequent oxidative stress upregulates cellular damage and suppress the functional activities of all other cells, while the build-up of impaired mitochondria further exacerbates the redox imbalance (Zhang J. et al., 2025b). However, the influx of mtDNA into the cytosolic space triggers cGAS-STING signaling pathways and upregulates inflammatory signaling mediated by NF-κB that predisposes to chronic-low grade inflammation and the inflammaging phenomenon (Xiao et al., 2023).

Nonetheless, the simultaneous reduction in NAD^+^-dependent deacetylases suppresses mitochondrial antioxidative capacity and disrupts the stress resistance capacity of FOXO (Son et al., 2025). Also, overstimulation of key pathways, including mTOR signaling, inhibits mitophagy and autophagy, while disrupting mitochondrial histology, hence promoting redox imbalance (Kong et al., 2025). Furthermore, mitochondrial dysfunction contributes to several signs of aging, such as chronic inflammation, cellular aging, stem cell wear-out, and reduced regenerative activity, while age-related conditions, including neurodegenerative disorders, metabolic dysfunction, sarcopenia and frailty, are observed at the organismal level, thus highlighting the potential role of mitochondrial dysfunction in accelerating age-related impairments (Jagaraj et al., 2024).

## 5 Therapeutic targeting of mitochondria

Since mitochondrial dysfunction contributes to the pathology of several human pathologies, including neurodegeneration, heart failure, inflammatory and metabolic conditions, they are considered an important target in the management of these disorders (Singh et al., 2021). The approach for targeting mitochondria was developed nearly 50 years ago, but only in the last decade has it started to become widely used for drug delivery (Zinovkin and Zamyatnin, 2019). Rather than managing the side effects of mitochondrial failure, molecular pharmacology and bioengineering aims to fundamentally restore homeostasis to the cellular energy system. These approaches take different forms, including the precise delivery of bioengineered antioxidants, pharmacological modulation of mitochondrial biogenesis and network dynamics, and genomic editing of mutated mtDNA (Zong et al., 2024) (Table 1).

**TABLE 1:** Therapeutic targeting of the mitochondria.

Therapeutic strategy	Mechanism of action	Key examples/Agents	Outcome/Goal
5.1 antioxidants and ROS modulators	Covalent linking of antioxidant moieties to lipophilic cations (e.g., TPP^+^) enables mitochondrial matrix accumulation driven by negative membrane potential, allowing targeted scavenging of mtROS.	MitoQ (ubiquinone derivative), SkQ1 (plastoquinone derivative)	Neutralizes superoxide, peroxyl, and peroxynitrite radicals; prevents lipid peroxidation; preserves electron transport chain function; reduces ROS-induced apoptosis
5.2 mitochondrial biogenesis and dynamics modulation	Upregulation of PGC-1α via AMPK/SIRT1 activation promotes mitochondrial biogenesis; inhibition of excessive Drp1-mediated fission restores mitochondrial network integrity	AICAR (exercise mimetic), Drp1 inhibitors, Mfn2 stimulants	Increases mitochondrial mass; improves energy homeostasis; prevents bioenergetic collapse and apoptotic signaling under stress
5.3 gene and mRNA therapies	Engineered nucleases selectively cleave mutant mtDNA in heteroplasmic cells, allowing repopulation with wild-type mtDNA; mRNA therapies import molecules to address mitochondrial protein/RNA deficiencies	mtZFNs, mitoTALENs	Eliminates pathogenic mtDNA mutations; restores OXPHOS function and normal mitochondrial membrane potential
5.4 mitochondrial transplantation and replacement	Transfer of functional exogenous mitochondria into damaged cells via tunneling nanotubes, extracellular vesicles, gap junctions, or endocytosis, integrating into host mitochondrial networks	Mitochondria isolated from healthy donor cells (e.g., skeletal muscle)	Improves cellular energy metabolism; restores mitochondrial function; prevents apoptosis; enhances recovery in ischemic injury
5.5 Small molecules and natural compounds	Raising NAD^+^ levels activates sirtuins to reduce ROS and promote fusion; AMPK/Nrf2 activation enhances antioxidant defenses and biogenesis; polyphenols modulate oxidative stress and calcium signaling	NAD^+^ precursors (NMN, NR, NAM), metformin, resveratrol, green tea polyphenols	Restores NAD^+^ homeostasis; halts ROS production; prevents mitochondrial fragmentation; stimulates biogenesis; protects against oxidative stress

### 5.1 Antioxidants and ROS modulators

While ROS play key role in signaling molecules at baseline levels, mitochondrial dysfunction causes pathological ROS overproduction, slowly becoming an underlying pathology in cellular damage (Zinovkin and Zamyatnin, 2019). Non-targeted antioxidants have largely failed in clinical trials for mitochondria-driven diseases because they do not penetrate the mitochondrial inner membrane to reach the matrix, which is exactly where ROS are being generated (Fields et al., 2023). To overcome this, targeted antioxidants have been developed and are typically synthesized by covalently linking an antioxidant moiety to a lipophilic cation, e.g., triphenylphosphonium (TPP+) (Jiang et al., 2020). Since the mitochondrial matrix has a highly negative membrane potential, it attracts the positively charged TPP+ and drives through the membrane, concentrating it hundred times higher in the mitochondria than in the cytoplasm (Kulkarni et al., 2021; Fields et al., 2023).

MitoQ is a ubiquinone derivative attached to the TPP cation by a 10-carbon aliphatic chain (James et al., 2007). It gets localized in the inner mitochondrial membrane and prevents lipid peroxidation by scavenging superoxide, peroxyl and peroxynitrite radicals (Sacks et al., 2021). Another drug, named SkQ1, is a derivative of plastoquinone conjugated to TPP+. SkQ1 has been found to form a complex with cardiolipin in order to prevent lipid peroxidation and ROS-induced apoptosis (Jiang et al., 2020). By neutralizing mtROS, these targeted antioxidants preserve the ETC and regulate mitochondrial metabolism against chronic oxidative damage (Fields et al., 2023). In a phase II clinical trial, SkQ1 has been demonstrated to be effective in the treatment of dry eye (Petrov et al., 2016). Plotnikov et al. (2013) also showed that SkQR1 effectively curtailed pyelonephritis by protecting mitochondrial integrity and lowering kidney damage via attenuating mitochondrial ROS in an *in vitro* and *in vivo* model. More so, SkQ1 delayed sarcopenia-associated damage of mitochondrial ultrastructure in Wistar and OXYS rat models (Vays et al., 2014) by alleviating age-dependent oxidative skeletal muscle damage. SkQ1 has also been shown to be useful in neurological disorders. Genrikhs et al. (2015) demonstrated that SkQ1 decreases trauma-induced neurological deficit and prevents amyloid-β-induced impairment of long-term potentiation via attenuation of mitochondrial oxidative stress in rat hippocampal slices.

#### 5.1.1 Clinical trial failures and limitations of targeted antioxidants

Despite promising data from preclinical studies, the clinical translation of targeted antioxidants has encountered substantial obstacles. The MitoQ Phase II trial in Parkinson’s disease (NCT00329056) did not meet its primary endpoint of slowing disease progression since it did not show any significant difference in Unified Parkinson’s Disease Rating Scale (UPDRS) scores between treatment and placebo groups (Snow et al., 2010). Similarly, a Phase II trial of MitoQ for hepatitis C (NCT00433108) showed no improvement in hepatic function markers or viral clearance (Gane et al., 2010). In amyotrophic lateral sclerosis (ALS), a Phase II trial of MitoQ (NCT00314015) was terminated early due to lack of efficacy and tolerability issues (Kaufmann et al., 2009). These failures may be attributable to several factors. First, there may be insufficient mitochondrial accumulation in target tissues, especially in the brain, where the blood-brain barrier limits TPP + conjugate penetration. Also, there may be inadequate potency against the high flux of mitochondrial ROS, requiring suprapharmacological concentrations that may be toxic. More so, this may be due to failure to address the primary cause of ROS overproduction; antioxidants scavenge ROS but do not repair the defective, ETC., that generates them. Furthermore, the redox paradox, excessive neutralization of ROS may disrupt physiological redox signaling, which is essential for cellular adaptation and stress responses (Akhigbe, 2025). These failures highlight that successful mitochondrial antioxidant therapy requires not only efficient delivery but also restoration of upstream bioenergetic defects.

### 5.2 Mitochondrial biogenesis and dynamics modulation

**FIGURE 2:**
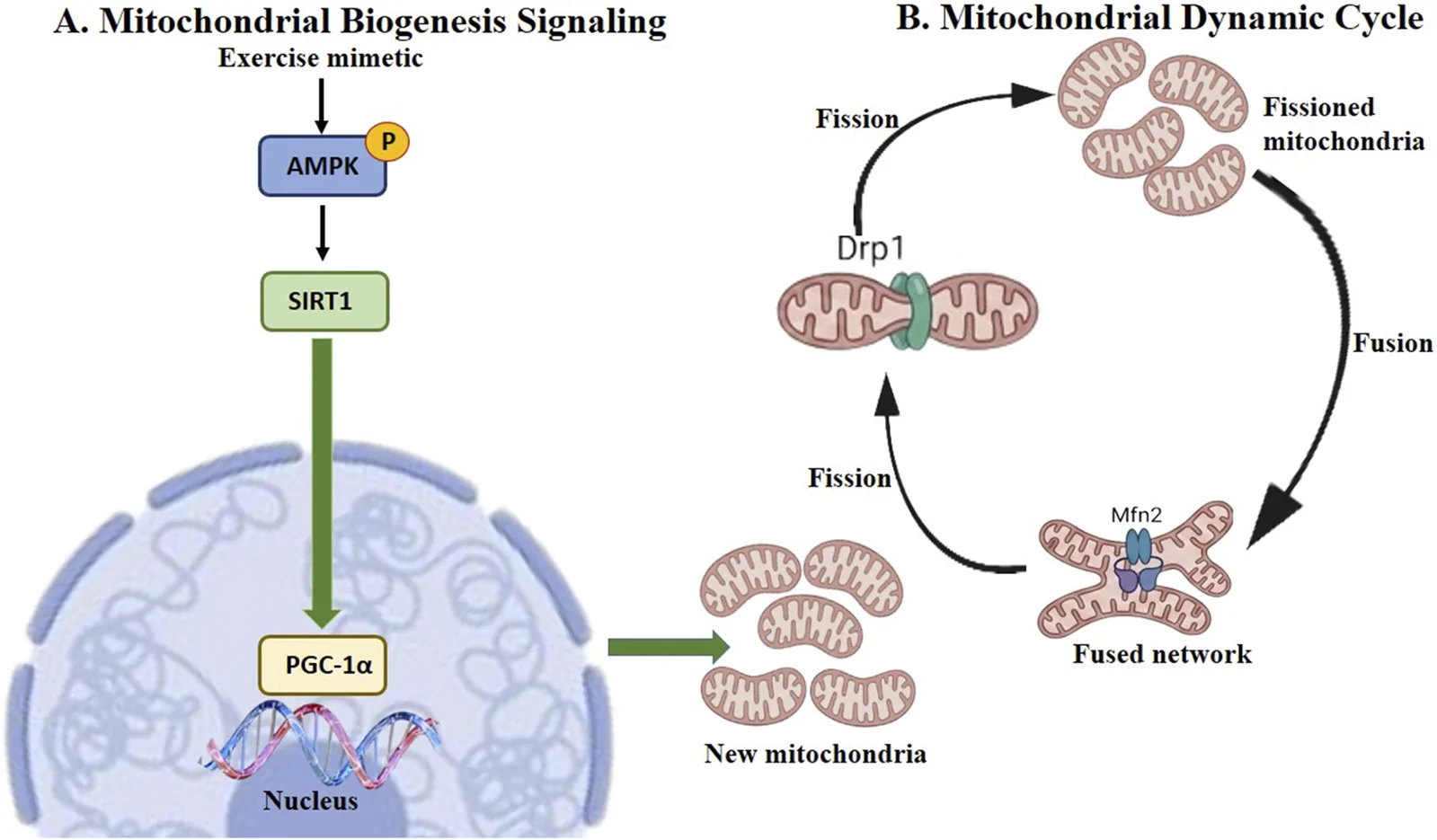
Mechanisms of pharmacological mitochondrial biogenesis and network dynamics. **(A)** Biogenesis signaling pathway illustrating how exercise mimetics (e.g., AICAR) activate the AMPK/SIRT1 axis, leading to the upregulation of PGC-1$\alpha$ and a subsequent increase in mitochondrial mass. **(B)** The ongoing cycle of mitochondrial dynamics, with Mfn2 fusion to interconnected networks and Drp1 fission to fragmented organelles.

The pharmacological strategies for the production of healthy mitochondria that can help to restore energy homeostasis are slowly becoming a center of focus (Singh et al., 2021) (Figure 2). Mitochondrial biogenesis refers to the process of increasing mitochondrial mass in cells. The transcriptional coactivators related to this process are the peroxisome proliferator-activated receptor γ-coactivator-1 (PGC-1) family, PGC-1α and PGC-1β (Ding et al., 2021). In metabolic disorder, where cells do not generate sufficient energy, it is a promising strategy to stimulate the upregulation of the PGC-1α via drugs (Popov, 2020).

This strategy involves the production of exercise mimetics, to replicate the systemic metabolic benefits of exercise (Tanaka et al., 2020). Mimetics like 5-aminoimidazole-4-carboxamide-1-β-D-ribofuranoside (AICAR) stimulate AMPK/SIRT1 axis, which deacetylates and activates PGC-1α, leading to improved mitochondrial biogenesis without physical effort (Halling and Pilegaard, 2020). In addition to mitochondrial biogenesis, therapeutic approach must address the regulation of mitochondrial dynamics, which is a physiological process that, through coordinated fusion and fission events, coordinates proper intracellular positioning of mitochondria at areas of high-energy requirements (Singh et al., 2021).

In diseased cells, these dynamics get dysregulated and sometimes undergoes excessive mitochondrial fission, mediated by pro-fission protein dynamin related protein 1 (Drp1) (Tanaka et al., 2020). Both inhibitor of fission and stimulants of fusion like protein mitofusin 2 (Mfn2), aim to revive an interactive mitochondrial network, thereby preventing bioenergetic collapse and apoptotic signaling in tissues under stress (Ding et al., 2021; Singh et al., 2021).

### 5.3 Gene and mRNA therapies

Mitochondrial mutations cause a number of debilitating disorders in the mitochondrial, and also contribute to many other complex diseases such as cardiovascular disease, cancer, and ageing, as well as neuromuscular and metabolic diseases (Zekonyte et al., 2020). There are about thousands copies of mtDNA in each human cell, which can lead to a condition called heteroplasmy, where both wild-type and mutant mtDNA molecules co-exist within the cell (Soldatov et al., 2022). Mutated mtDNA only has negative effect when a threshold of mutant load occurs (Bacman et al., 2020). This mutant fraction can however be eliminated selectively to restore OXPHOS function and mitochondrial membrane potential (Nissanka and Moraes, 2020).

CRISPR-Cas9, a gene therapy, is a type of nuclear DNA editing that is widely known and used, but mtDNA editing has not been successful due to several limitations, including the difficulty in delivering the single-guide RNAs (sgRNAs) into human mitochondria (Wang et al., 2026). To overcome this particular limitation, engineered nucleases like, mitochondria-targeted zinc finger nucleases (mtZFNs) and transcription activator-like effector nucleases (mitoTALENs) are being adapted (Bacman et al., 2020). The nucleases are designed to recognize specific sequences in mtDNA, *in vitro* and *in vivo*, thus expanding the number of mtDNA mutations that can be targeted (Zekonyte et al., 2020). This system allows for selective cleavage and once depletion of mtDNA is induced in the transfected cells or infected tissues, the mutant mtDNA are degraded and the cells are quickly repopulated with the wild-type mtDNA. These steps have a great potential for therapeutic application in mtDNA disorders (Bacman et al., 2020). Furthermore, new mRNA therapies aim to import amino acids or nucleotide residues directly into the matrix of mitochondria using special natural or artificial transporters, for the purpose of addressing deficiencies in mitochondrial proteins or RNA (Soldatov et al., 2022).

#### 5.3.1 Delivery bottlenecks and off-target challenges for mtDNA editing

Although mitochondrial gene-editing technologies are promising, they are faced with translational barriers that have prevented clinical application. Viral vector delivery limitations represent a critical bottleneck. Adeno-associated virus (AAV) vectors, the most widely used gene delivery platform, have a packaging capacity of approximately 4.7 kb, which is insufficient for most mitoTALEN and mtZFN constructs (typically 5–8 kb) when combined with mitochondrial targeting sequences and promoter elements (Bacman et al., 2024). Dual-vector strategies (split-intein or overlapping AAVs) have been explored but suffer from low co-transduction efficiency, poor expression of reconstituted proteins, and increased immunogenicity (Zobel, 2022). Moreover, AAV transduction of post-mitotic tissues like neurons, cardiac myocytes, and skeletal muscle is inefficient, requiring high vector doses that trigger dose-dependent hepatotoxicity and complement activation (Wu et al., 2025). Nucleic acid delivery platforms such as lipid nanoparticles (LNPs), which have succeeded for mRNA vaccines, face unique difficulties with mitochondrial delivery (Liew et al., 2021). Furthermore, the vast majority of LNP formulations accumulate in the liver and spleen, limiting their utility for neurological or cardiac indications. Off-target cleavage and genotoxicity constitute another major concern. Double-strand breaks in mtDNA can trigger mtDNA degradation without wild-type repopulation if the heteroplasmic mutant load is extremely high, potentially precipitating mitochondrial depletion syndrome (Nissanka and Moraes, 2020). Finally, immune responses against bacterial-derived nucleases may trigger pre-existing or *de novo* humoral and cellular immunity in humans, limiting repeat dosing and long-term efficacy. These interconnected bottlenecks collectively impede the clinical translation of mtDNA editing technologies and underscore the need for improved delivery systems, alternative nuclease designs with enhanced specificity, and rigorous preclinical safety testing in humanized animal models.

### 5.4 Mitochondrial transplantation and replacement

**FIGURE 3:**
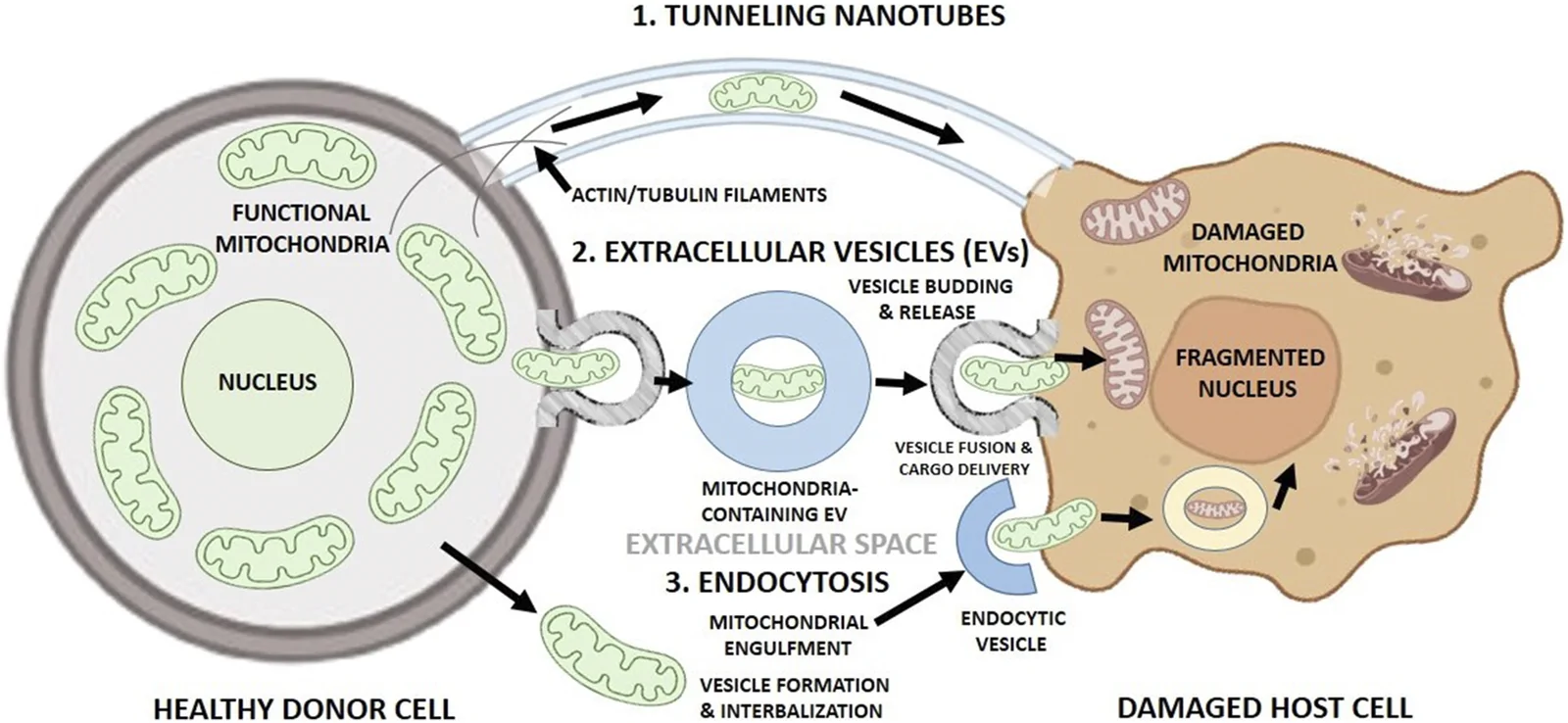
Mechanisms of intercellular mitochondrial transfer and transplantation. In mitochondrial transplantation, healthy mitochondria are incorporated into the host cells through **(A)** tunneling nanotubes, **(B)** extracellular vesicles (EVs), and **(C)** gap junctions, or the exocytosis and endocytosis of free mitochondria depending on the type and conditions of cells.

Mitochondrial transplantation has emerged as a new promising option for the treatment of mitochondrial diseases caused by mtDNA mutations. This procedure involves damaged cells engulfing and incorporating mitochondria from another cell with a viable function, so as just to improve biological processes (Park et al., 2021; D’Amato et al., 2023). The process can take place through tunneling nanotubes (TNTs), extracellular vesicles (EVs), gap junctions, or the exocytosis and endocytosis of free mitochondria depending on the type and conditions of cells (Sendra et al., 2021) (Figure 3).

Preclinical and clinical trials have shown that mitochondrial transplantation can improve cellular metabolism, restore mitochondrial function, and inhibit apoptosis (Kim et al., 2023). A healthy skeletal muscle cells can be used as mitochondria donors to cardiomyocytes without side effects. Also, it has been shown that mitochondria isolated and transplanted into the ischemic zone of the myocardium can boost up recovery in cardiac ischemia-reperfusion injury (Ali-Pour et al., 2020). Kanai et al. (2025) visualized and quantified mitochondrial internalization by mesenchymal stromal cells and found that mitochondrial uptake was an active energy-dependent process mediated by multiple endocytic pathways.

The internalized mitochondria are incorporated into host cells, requiring factors such as the metabolic status of the cells (Kim et al., 2023). One of the main challenges is the mitochondrial long-term storage upon isolation. In addition, delivery of functional mitochondria to the right cells within the body, and the stability and activity of the mitochondria over time, are important issues that need to be explored (D'Amato et al., 2023). Once these conditions are met, mitochondrial transplantation could be designed to correct specific mutations with the unprecedented advantage of having a broad applicability in various mitochondrial diseases.

### 5.5 Small molecules and natural compounds

Small molecules play an essential role in drug development and are beneficial to enhance mitochondrial function. Raising cellular NAD+ (Nicotinamide adenine dinucleotide) through vitamin B3 family precursors or via drug-based interventions has become a largely used option to restore mitochondrial and organismal homeostasis, with NAD + precursors becoming a popular supplementation approach (Yusri et al., 2025). NAD + precursors, such as nicotinamide (NAM), nicotinamide mononucleotide (NMN) and nicotinamide riboside (NR), can help control the activity of sirtuins (Poljšak et al., 2023). Focusing on sirtuins, recent studies consider their activation as a protective one as it helps halt ROS production, prevent mitochondrial fragmentation, and promote mitochondrial fusion (Poljšak et al., 2023).

Metformin, an antidiabetic drug, complements sirtuin actions by inhibiting complex I of the mitochondrial respiratory chain (Saberi et al., 2021). It is also a potential activator of AMPK and stress-induced transcription factor, nuclear factor erythroid 2-related factor 2 (Nrf2), that has potential to treat mitochondrial diseases (Meng and Wu, 2023). There are also numerous natural polyphenols, such as resveratrol and green tea polyphenols, which have the ability to alter the pathways of oxidative stress and Ca^2+^ signaling, and promote expression of mtDNA by activation of PGC-1α, which stimulates mitochondrial biogenesis (Guan et al., 2025).

### 5.6 Translational barriers and strategies for clinical implementation

Beyond the specific limitations of distinct therapeutic modalities, several cross-cutting translational challenges impede the clinical development of mitochondrial therapies. First, the lack of robust biomarkers for mitochondrial dysfunction hinders patient stratification and real-time monitoring of therapeutic response. Traditional markers such as serum lactate, pyruvate (Saka et al., 2026a; Saka et al., 2026b), and fibroblast growth factor 21 (FGF21) lack sensitivity and tissue specificity (Shayota, 2024). Additionally, the heterogeneity of mitochondrial diseases, with hundreds of distinct mtDNA and nuclear mutations, makes it difficult to design therapies that benefit broad patient populations (Pitceathly et al., 2021). Individualized or mutation-specific strategies will likely be required, which are economically challenging and logistically complex. Moreover, the blood-brain barrier remains a formidable obstacle for central nervous system applications, as most mitochondrial drugs, nucleic acids, and enzymes are large, hydrophilic, or charged molecules that do not cross the BBB. Furthermore, the incomplete understanding of mitochondrial tissue-specific pathobiology means that interventions effective in one organ may be ineffective or detrimental in another. Finally, regulatory challenges are significant: mitochondrial transplantation, gene editing, and organelle-targeted therapies fall into novel regulatory categories for which frameworks are still evolving, requiring extensive safety and efficacy data before first-in-human trials can proceed.

Despite these obstacles, emerging approaches offer promising opprotunities. Nanoparticle-based delivery systems with mitochondrial targeting moieties, such as TPP-conjugated polymers and mitochondriotropic peptides, are being optimized to enhance biodistribution and cellular uptake (Li et al., 2024). Also, blood-brain barrier-penetrant transporters such as the transferrin receptor or low-density lipoprotein receptor (LDLR)-mediated transcytosis are being exploited to deliver mitochondrial therapeutics to the brain (Tang et al., 2026). Combined approaches that concurrently address multiple pathogenic mechanisms like ROS, bioenergetics, dynamics, and mtDNA integrity may offer synergistic benefits.

## 6 Conclusions and future directions

Mitochondria have evolved far beyond their familial role as ATP generators. These organelles function as cellular centres that integrate energy metabolism, calcium homeostasis, redox signalling, and programmed cell death. Their dynamic nature, manifested via coordinated series of biogenesis, fusion, fission, and mitophagy, enables precise adaptation to physiological demands across diverse tissues. Consequently, mitochondrial integrity is essential to cellular and organismal health.

Mitochondrial dysfunction, arising from bioenergetic failure, genetic mutations, or oxidative stress, is implicated in several pathologies, such as metabolic syndrome, neurodegenerative disorders, cardiovascular disease, reproductive dysfunction, cancer, and ageing. This review has systematically highlighted the molecular mechanisms of mitochondrial function and dysfunction, linking mitochondrial impairment to disease pathogenesis and exploring emerging therapeutic strategies.

The therapeutic landscape has expanded considerably. Targeted antioxidants (MitoQ, SkQ1) neutralise pathological ROS at their source; PGC-1α activators and NAD^+^ precursors rejuvenate mitochondrial networks; gene-editing technologies (mitoTALENs, mtZFNs) offer potential to eliminate pathogenic mtDNA mutations; and mitochondrial transplantation represents a paradigm-shifting approach to organelle replacement. Yet clinical translation is limited. The failure of MitoQ in Phase II trials for Parkinson’s disease, hepatitis C, and ALS underscores the gap between preclinical promise and clinical reality, highlighting inadequate BBB penetration, lack of robust biomarkers, and failure to address upstream bioenergetic defects rather than downstream ROS.

Future research must prioritize overcoming delivery barriers, especially the blood–brain barrier, through receptor-mediated transcytosis, extracellular vesicles, and focused ultrasound. Tissue-specific targeting using engineered lipid nanoparticles and mitochondriotropic peptides is vital. Regulatory challenges, including the lack of validated surrogate endpoints, disease heterogeneity, and evolving frameworks for gene-editing and mitochondrial replacement, need collaborative regulatory science and adaptive trial designs. Ethical considerations surrounding germline modification, identity, equity, and informed consent require transparent governance and public engagement. Improved preclinical models, including patient-derived iPSCs and organoids, and combination therapies addressing multiple pathogenic mechanisms simultaneously, are critical for successful translation. The future of mitochondrial medicine does not lie in a single “magic bullet” but in the integration of sophisticated delivery science, rigorous regulation, thoughtful ethics, and interdisciplinary collaboration, eventually transitioning from symptomatic management to restoration of mitochondrial health.
